# Activation of Brain Attention Systems in Individuals with Symptoms of ADHD

**DOI:** 10.1155/2007/865717

**Published:** 2007-05-30

**Authors:** P. Dennis Rodriguez, Gordon C. Baylis

**Affiliations:** ^1^Indiana University South BendINUSA; ^2^University of South CarolinaColumbiaSCUSA

**Keywords:** ADHD, ADHD subtypes, ADHD symptoms, attention-deficit/hyperactivity disorder, ERP, Go/NoGo task

## Abstract

Previous research investigating attention and impulse control in individuals with Attention-Deficit/Hyperactivity Disorder (ADHD) has largely ignored the symptomatic differences among the three subtypes of ADHD: ADHD-Inattentive Type, ADHD-Hyperactive/Impulsive Type, and ADHD-Combined Type. The present study examined attention and impulse control by focusing on these subtypes. Based on their self-reported symptoms of ADHD, participants belonged to one of four groups: ADHD-Inattentive, ADHD-Hyperactive/Impulsive, ADHD-Combined, and control. Cortical activity was recorded from participants during performance of a Go/NoGo task. The event-related potentials (ERP) measured at frontal and posterior sites discriminated between the control group and participants with symptoms of ADHD. The control group consistently exhibited a higher P3 amplitude than all the ADHD groups. The main difference occurred at the frontal site, indicating that individuals with ADHD symptoms have deficits in the anterior attentional system, which mediates signal detection. Behavioral measures of signal sensitivity revealed that the ADHD-Inattentive and the ADHD-Hyperactive/Impulsive groups had more difficulty with the attention-demanding Go/NoGo respond-to-target task, while behavioral measures of response bias indicated that the ADHD-Hyperactive/Impulsive and the ADHD-Combined groups responded more liberally in the inhibition-demanding Go/NoGo suppress-to-target task.

## 1. Introduction

Attention-Deficit/Hyperactivity Disorder (ADHD) has largely been viewed as a childhood disorder, although it is also prevalent in the adult population [1]. ADHD is characterized by "a persistent pattern of inattention and/or hyperactivity-impulsivity that is more frequent and severe than is typically observed in individuals at a comparable level of development" [2, p. 78]. Many studies have used individuals with ADHD to better understand the biological and neural aspects of attention but have largely ignored the symptomatic differences among the three subtypes of ADHD: ADHD-Inattentive Type, ADHD-Hyperactive/Impulsive Type, and ADHD-Combined Type. These subtypes do not exhibit the same pattern of behaviors; therefore, some consider the underlying deficits to vary across subtypes [1]. The present study focuses on these ADHD subtypes to determine neurological and behavioral differences between them by recording their scalp activation and assessing their performance during tasks that demand attention and impulse control in young adults.

Some argue that individuals with ADHD have difficulty sustaining, focusing, or shifting their attentional resources [3]. Others propose that individuals suffering from ADHD have difficulty responding to attended stimuli [4]. Although most individuals diagnosed with ADHD display symptoms of both inattention and hyperactivity/impulsivity (so called Attention-Deficit/Hyperactivity Disorder, Combined Type [2]), there are others in whom one pattern is more predominant, resulting in one of the following: Attention-Deficit/Hyperactivity Disorder, Pre-

ISSN 0953-4180/07/$17.00 © 2007 – IOS Press and the authors. All rights reserved dominantly Inattentive Type (ADHD-IA), or Attention-Deficit/Hyperactivity Disorder, Predominantly Hyperactive/Impulsive Type (ADHD-HI). However, Milich, Balentine, and Lynam [5] argued that the ADHD-IA and ADHD-Combined types are unrelated disorders and do not share the same characteristics currently described in their DSM-IV classification. The present study, though, adheres to the DSM-IV classification of ADHD but addresses the Milich et al. [5] study later.

One task frequently used to detect attentional dysfunction is the Continuous Performance Test, or CPT [6–9]. The CPT is a well-established behavioral task adopted to investigate sustained attention. Stimuli are presented on a computer screen, and participants analyze the stimuli, determine if the stimuli are targets or distracters, and respond accordingly on a mouse or keypad [10]. Central to the CPT are errors of omission (misses) and errors of commission (false alarms). Omission errors are believed to indicate inattention, while commission errors are thought to index impulsivity [11]. The CPT differs from the Go/NoGo task, in one crucial way: a Go/NoGo task involves execution and inhibition of a prepared motor response, whereas the CPT can involve execution and inhibition as well as differential responding [10]. For example, in the Defrance et al. [11] CPT, the differential response consisted of pressing the right mouse button in some cases and pressing the left mouse button in others. In a Go/NoGo task, only one of these two responses is required, taking away the differential responding and leaving only the execution and inhibition of a prepared motor response. Studies using different versions of CPT or Go/NoGo tasks have found that hyperactive and children with ADHD make more errors (e.g. [12,13]) and have slower reaction times than controls (e.g. [12]). Children with ADHD consistently demonstrate inferior performance levels on tests of persistence and sustained attention [3], which are features of the Go/NoGo task. Therefore, the present study uses a Go/NoGo task.

A number of studies analyzed event-related potentials (ERP [14,15]) to study the brain basis of ADHD. ERPs are comprised of several different waveforms, with earlier positive waveforms (P1 and P2) thought to relate to processing of the physical attributes of the stimulus. The early negative waveforms (N1 and N2) seem to reflect other aspects of stimulus processing, such as feature analysis. The later positive waveforms reflect judgmental processes, independent of the physical aspects of the stimuli [11,16]. The present study investigates one such late positivity termed P3 (occurring approximately 300 ms after stimulus onset), for the P3 is associated with the identification processes related to the detection of task-relevant stimuli [17].

The P3 reflects multiple cognitive processes, specifically attentional resource allocation [18]. P3 amplitude is thought to be a reflection of the effortfulness of the stimulus response and the intensity of processing, whereas P3 latency is taken as a reflection of the speed of information processing [19]. Some have found that P3 latency increases as categorization of the stimulus becomes more difficult [20–22] while others have reported that P3 latency remains unaffected by task difficulty [23]. The present study is interested in how the effortfulness of processing is reflected in the different subtypes of ADHD by examining P3 amplitude. The P3 component is most pronounced when a stimulus is unpredictable, task relevant, related to response selection, or interfering with attention [9,24]. When a task is difficult, however, more effort is needed to maintain a constant level of performance than in an easy task, and the brain structures involved are not activated in local synchrony, leading to a diminished P3 amplitude [20, 22]. The attention allocated to a task is also under intentional control, and therefore, an individual's approach to the task may affect the P3 [22]. This may mean that the P3 is less sensitive to increased task demands than behavioral measures [25]. P3 amplitude, however, decreases as memory load increases [22]. Furthermore, it is possible that individuals with ADHD have fewer resources available. This suggests that individuals with ADHD may show reduced P3 amplitudes on the same Go/NoGo task as controls [26].

The P3 component can be divided into the anterior P3a and the posterior P3b. The P3a component has a shorter peak latency than the P3b, habituates rapidly, and is thought to reflect frontal lobe function [27,28], while the P3b is thought to reflect parietal lobe function [29]. The P3a is generally conceived as an alerting response that most likely originates from neural sources related to initial attention allocation [20,30]. The P3b, however, reflects the match between the stimulus and an internal representation of a target [31]; see also [33]. Harmony et al. [20] noted that both the P3a and the P3b are inversely related to task demands (the greater the difficulty, the more suppressed the amplitude), indicating that although they reflect different cognitive processes, they are manifestations of what is generally termed the P3. This view, however, is debated; for a review see Fabiani et al. [32]. For the purposes of the present study, the term P3a refers to the P3 component having an anterior scalp distribution, and the term P3b refers to the P3 component having a posterior scalp distribution.

Kemner and colleagues [34,35] found that children with ADHD exhibited smaller P3 amplitudes than controls at Pz in response to auditory deviant stimuli, while autistic and dyslexic children did not show such a deficit. Similarly, Verbaten et al. [36] found a reduced frontal P3 in children with ADHD (see also [4, 12]). An explanation given by Miller et al. [3] for the reduced P3 amplitude exhibited by individuals with ADHD is that they have to allocate greater attentional resources in the later stages of information processing in order to maintain a level of performance equal to normal controls. In terms of the latency of the P3, a few studies have reported increased P3 latencies in children with ADHD [37–39], most found no significant differences [40], while one study [41] found that controls had significantly longer latencies than participants with ADHD. Clearly, consensus does not exist on P3 latency in individuals with ADHD.

Winsberg et al. [9] suggested that P3 deficits are mainly detectable under conditions that elicit performance deficits, such as the Go/NoGo task. The ERPs elicited by Go and NoGo targets contain a pronounced P3 in healthy controls. Fallgatter et al. [10] showed that the positive area of the P3 fields was more anterior when associated with an inhibition than with an execution, terming this the NoGo-anteriorization (NGA). Fallgatter et al. [10] concluded the NGA is due to the relative contribution of inhibitory frontal lobe activity. Increased amplitude and latency of the P3 in the NoGo condition was interpreted as an indicator of higher processing demands in that condition [42,43]. If the different clinical classification of ADHD subtypes reflects different underlying failures of attention and inhibition, one would predict that the Go/NoGo task would for the discrimination between subtypes electrophysiologically as well as behaviorally depending on the response and suppression demands of the task. This prediction is tested directly for the first time in the present study.

The current study uses a Go/NoGo task to elicit electrophysiological differences and performance deficits due to attention and impulse control in ADHD-symptomatic individuals. These individuals were chosen based on self-reported measures described below and fall into four categories: ADHD Inattentive Type (ADHD-IA), ADHD Hyperactive/Impulsive Type (ADHD-HI), ADHD Combined Type, and control. This study analyzed the peak amplitude and latency of the P3a and P3b components associated with the Go/NoGo targets, the reaction time to targets and distracters, and sensitivity and response bias to targets. Although the ADHD groups in the present study comprise individuals who self-reported symptoms of ADHD, it is likely that they share characteristics with members of the clinical population [44].

For the present study, we found the differences between controls and individuals with ADHD by comparing individuals with ADHD as a whole to controls without considering the different symptoms associated with ADHD. This is done to ensure that the current study provides findings consistent with past research. Once this is established, we shift our focus to the subgroups of ADHD and hypothesize firstly that individuals with ADHD-IA or ADHD-Combined will exhibit a smaller P3 amplitude than controls on the respond-to-target task and that they will have less sensitivity to these stimuli than controls. The second hypothesis states that individuals with ADHD-HI and ADHD-Combined will show reduced P3 amplitude compared to controls in the suppress-to-target task and that they will also have lower sensitivity to these stimuli than controls.

## 2. Method

### 2.1. Participants

A priori power estimation from pilot data revealed that setting power at 0.80 and $\alpha = 0.05$, a per group $n$ of 16 was needed to detect a medium effect size. Therefore, 64 undergraduate students participated initially. The sample consisted of 34 females and 30 males (ratio = 1.14:1) with normal or corrected-to-normal vision. Their ages ranged from 18 to 24 ($M = 19.5$, $SD = 1.94$). Fifty-seven participants were right-handed, 5 were left-handed, and 2 reported no handedness preference. Eighteen participants reported being medically diagnosed with ADHD and having no history of other neurological or psychological problems. The remaining participants reported no neurological or psychological problems. Nine participants were currently taking medication for the treatment of ADHD (Adderall or Ritalin) but had not taken their usual dose during 12 to 24 hours before testing. Because methylphenidate (Ritalin) and mixed amphetamine salts (Adderall) have a half-life of 3–4 hours and 4–6 hours respectively [45], researchers consider a 24-hour elimination period adequate [46], since only minuscule amounts of the drug remain in the blood beyond the 24-hour mark [47]. Of the nine medicated participants, only one reported a 12-hour elimination period; all others reported 24. Dropping this one participant from data analysis did not change the results; therefore, results reported in this paper reflect the inclusion of said participant. Two other participants were dropped from analysis because they were medicated at the time of testing, and 4 participants were dropped because their data did not reveal a distinguishable P3 component. However, six more participants replaced those dropped, for a total of 64.

We used three methods to recruit participants. We initially administered questionnaires that included the DSM-IV checklist for ADHD symptoms [48] to freshmen classes (see Materials section). Students voluntarily completed the questionnaires and voluntarily provided their phone number or electronic mail address. After analysis of the DSM-IV checklist, students were contacted via telephone or electronic mail and offered a monetary incentive to participate in the study. The second method of recruitment consisted of posting an advertisement asking students to contact the experimenter via electronic mail if they had received a clinical diagnosis of ADHD. These students were given monetary incentive to participate in the study. Finally, we also recruited participants from undergraduate psychology classes. These students completed Barkley's [48] checklist and were assigned to one of the conditions according to the criteria stated below and received course credit for their participation.

Participants scoring above 11 on the inattentive items scale but below 9 on the hyperactive/impulsive items scale were assigned to the ADHD-IA condition. Those scoring above 9 on the hyperactive/impulsive items scale but below 11 on the inattentive items scale were assigned to the ADHD-HI condition. Participants scoring above 11 on the inattentive items scale and above 9 on the hyperactive/impulsive items scale were assigned to the ADHD-Combined condition. The control group consisted of participants that scored 5 or below on both scales.

### 2.2. Materials

The DSM-IV checklist for ADHD symptoms [48] consists of 9 inattentive items and 7 hyperactive/ impulsive items. All inattentive and hyperactive/impulsive items are on a 4-point scale (not at all = 0, just a little = 1, pretty much = 2, very much = 3). The inattentive items scale ranges from 0 (no ADHD-IA symptoms endorsed) to 27 (all ADHD-IA symptoms endorsed as "very much"). The hyperactive/impulsive items scale ranges from 0 (no ADHD-HI symptoms endorsed) to 21 (all ADHD-HI symptoms endorsed as "very much"). Univariate analysis of the DSM-IV checklist for ADHD revealed that 95% of students scored below 11 on the inattentive items and below 9 on the hyperactive/impulsive items, based on normative data from 1,411 first year college students. These scores became the criteria for assigning participants into each condition, for it is presumed that ADHD is prevalent in 5% of the population [1]. Table 1 lists the mean items endorsed by each group for both the inattentive and the hyperactive/impulsive items.

Table 1
Mean ADHD Items Endorsed by Group

| Groups | Mean Inattentive Items Endorsed | Mean Hyperactive/ Impulsive Items Endorsed |
|---|---|---|
| Control | 3.06 (2.11) | 1.75 (1.71) |
| ADHD-IA | 15.18 (5.93) | 4.62 (3.83) |
| ADHD-HI | 6.43 (3.32) | 11.25 (3.83) |
| ADHD-Combined | 14.87 (5.18) | 15.87 (5.55) |

*Note*$_1$. *SD* listed in parentheses.
*Note*$_2$. ADHD-IA criteria: $>$ 11 inattentive items endorsed and $<$ 9 hyperactive/impulsive items endorsed. ADHD-HI criteria: $<$ 11 inattentive items endorsed and $>$ 9 hyperactive/impulsive items endorsed. ADHD-Combined criteria: $>$ 11 inattentive items endorsed and $>$ 9 hyperactive/impulsive items endorsed.

Participants sat 117 cm from a 29" color video computer monitor (NEC Multisync XM29) displaying at 1280 horizontal and 1024 vertical pixels. The visual stimuli consisted of black numbers from one (1) to nine (9) in three different sizes, Times New Roman 18 pt, 48 pt, and 78 pt, for a total of 27 stimuli. The small numbers measured 10 mm high and 7 mm wide, resulting in a visual angle of 4.90$^\circ$ vertically and 3.43$^\circ$ horizontally. The medium numbers measured 30 mm high and 20 mm wide, resulting in a visual angle of 9.77$^\circ$ vertically and 14.61$^\circ$ horizontally. The large numbers measured 50 mm high and 33 mm wide, resulting in a visual angle of 24.12$^\circ$ vertically and 16.05$^\circ$ horizontally. The order of presentation and the interstimulus interval were random. The stimuli appeared on screen for 1000 ms, and the interstimulus interval ranged from 550 ms to 950 ms.

This study consisted of two separate Go/NoGo tasks: a "respond-to-target" task, and a "suppress-to-target" task. In the respond-to-target task, the target stimuli (the medium-sized numbers 1, 2, and 3), which signaled response execution, were each presented 6% of the time during one block of trials, totaling a combined presentation of 18% (30 presentations) per block. Participants responded by pressing a key on a response box every time these target numbers appeared on the screen. In the suppress-to-target task, the target stimuli (the number 8 in all three sizes), which signaled response inhibition, were each presented 6% of the time during one block of trials, totaling a combined presen-

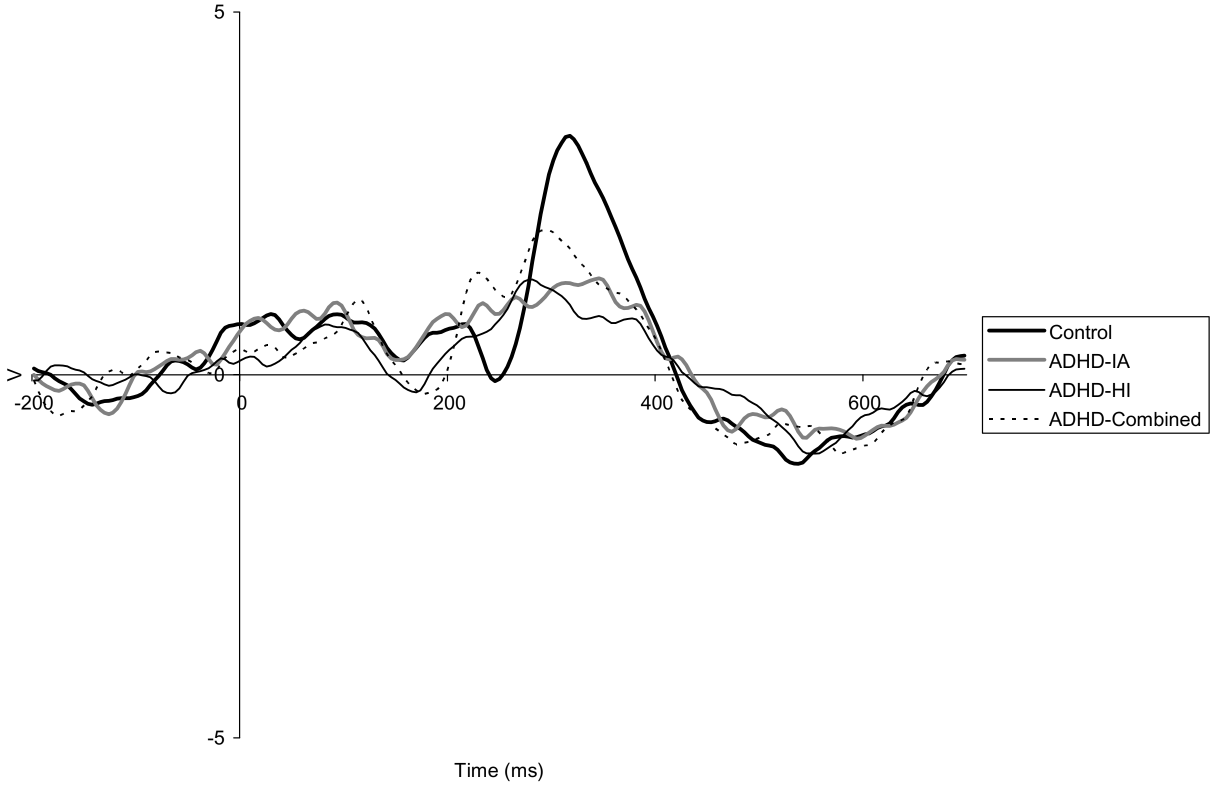

Fig. 1. Grand-average ERP waveforms for ADHD subgroups and controls at electrode 11 (comparable to Fz) on the respond-to-target task.

tation of 18% (30 presentations) per block. Participants responded by pressing a key on a response box to all numbers appearing on the screen except to the number eight, regardless of its size.

### 2.3. Procedure

Once the participants arrived for their testing session, they read and signed a consent form and completed a medical questionnaire in order for the experimenter to determine if the participants were currently using prescription medication aiding their concentration or memory, to determine if the participants had any other neurological or psychological disorders, to determine the participants' handedness, and to assess the participants' general health. Participants completed the ADHD checklist [48] for a second time and were assigned to one of the four conditions, ADHD-IA ($n = 16$), ADHD-HI ($n = 16$), ADHD-Combined ($n = 16$), and control ($n = 16$).

Participants had two practice sessions lasting one minute each. The first practice session consisted of the respond-to-target condition, and the other consisted of the suppress-to-target condition. Once the experimenter and the participant both were confident the participant understood the instructions, the experimenter left the room and the actual experiment began. Each block of trials for the respond-to-target and suppress-to-target tasks lasted 6 minutes. The experimenter then reentered the room and reminded the participant of the instructions for the next task. The blocks of trials for the respond-to-target and suppress-to-target tasks were presented three times each, with the order of presentation counterbalanced across the sample. Participants received a debriefing form once the study reached an end.

### 2.4. Electrophysiological data analysis

Scalp EEG was recorded through a sensor net, part of the Electrical Geodesics Incorporated High-Density EEG system [49] with amplifiers for collecting 128 channels of EEG data and high-impedance "geodesic electrodes" as transducers for the EEG. The impedance threshold was set at 100 k$\Omega$. The vertex served as the reference for the EEG signal, which was recorded at a sampling rate of 250 Hz (4 ms samples), and the common electrode was located at the nasion.

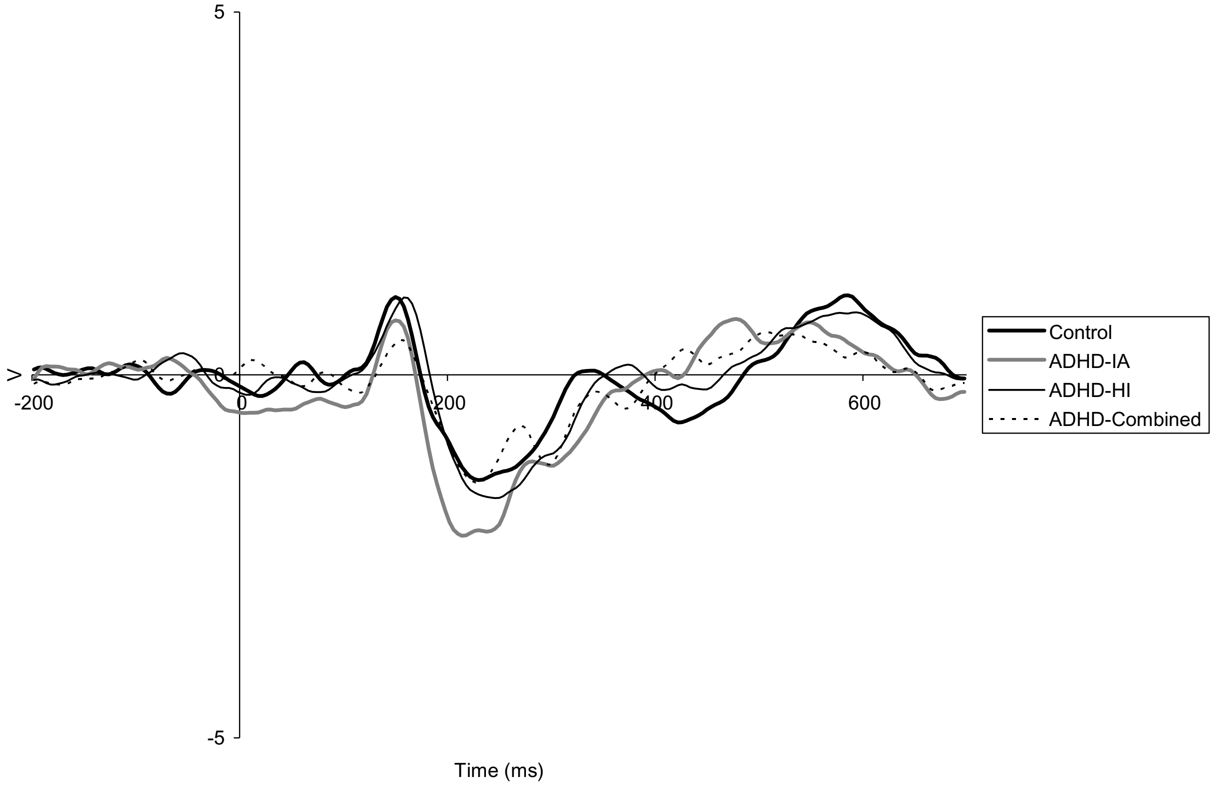

Fig. 2. Grand-average ERP waveforms for ADHD subgroups and controls at electrode 62 (comparable to Pz) on the respond-to-target task.

After recording, data were segmented using a 200 ms pre-stimulus interval and a 700 ms post-stimulus onset for correct respond-to-target and suppress-to-target trials separately. Segments were then averaged using EGI Analysis Tools [50] to derive ERPs for each participant. Based on the EGI guidelines [50], we eliminated trials containing more than 10% bad channels as well as trials containing an eye blink during the 900 ms segment. The bad channel algorithm detects bad channels by measuring the difference between fast and slow running averages of channel amplitude. Once these were detected, they were removed from the averaging procedure. Data were then filtered offline from 0.1–30 Hz and baseline corrected using the 200 ms pre-stimulus interval.

ERP analysis consisted of only correct responses (hits) for the respond-to-target task and only correct rejections for the suppress-to-target task. We measured the P3a at frontal electrode 11, comparable to Fz on the Jasper 10–20 system [51,52], and the P3b at parietal electrode 62, comparable to Pz on the Jasper 10–20 system [51,52]. A grand average ERP for all participants showed that the grand average P3a for the respond-to-target task occurred between 160–424 ms and for the suppress-to-target task between 168–388 ms. The grand average P3b for the respond-to-target task occurred between 448–660 ms and for the suppress-to-target task between 304–392 ms. We then identified the positive peak amplitude for each participant that occurred during these intervals as the respective P3a or P3b and used for the amplitude analysis. The latency for that peak was used for the latency analysis.

### 2.5. Behavioral data analysis

For each participant, we calculated $d'$ as a measure of stimulus sensitivity using Theory of Signal Detection and conducted a one-way ANOVA on $d'$ for levels of ADHD. In addition, we calculated $\log(\beta)$ as a measure of response bias and conducted a one-way ANOVA on $\log(\beta)$ for levels of ADHD. We also analyzed reaction times to the correct responses in the respond-to-target task and the false alarms in the suppress-to-target task with one-way ANOVAs.

### 2.6. Design for statistical analysis

For the purposes of this study, we consider factor ADHD as having four levels: control group

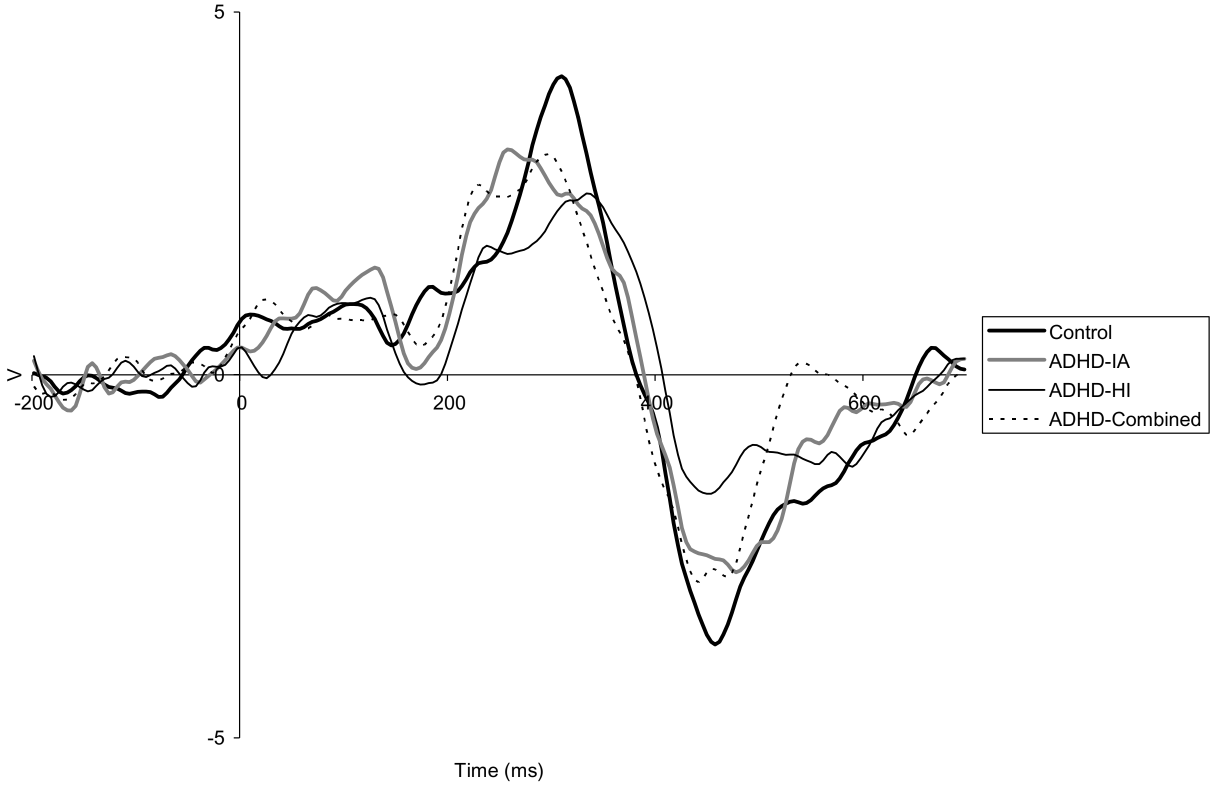

Fig. 3. Grand-average ERP waveforms for ADHD subgroups and controls at electrode 11 (comparable to Fz) on the suppress-to-target task.

(no ADHD symptoms), ADHD-IA (inattentive symptoms), ADHD-HI (hyperactive/impulsive symptoms), and ADHD-Combined (both inattentive and hyperactive/impulsive symptoms). Analyses of the effect of levels of ADHD on all dependent variables were conducted separately with one-way between-subjects ANOVAs. We then made comparisons between the control group and "ADHD-All" by collapsing data across ADHD-IA, ADHD-HI, and ADHD-Combined. We calculated these planned comparisons after finding the omnibus $F$ and using its error term. The purpose of the comparisons was to determine whether replication of previous studies was accomplished; therefore, we report them first followed by the $F$ statistic for the one-way between-groups ANOVA. We used post hoc Tukey HSD pairwise comparisons for analyses revealing a significant difference for levels of ADHD.

## 3. Results

Table 2 provides the means and standard deviations of the P3a and P3b amplitude and latency for the ADHD subtypes and controls on the respond-to-target task. The means and standard deviations of the P3a and P3b amplitude and latency for the ADHD subtypes and controls on the suppress-to-target task are presented in Table 3. Figures 1, 2, 3, and 4 depict grand-average ERP waveforms of ADHD subtypes and controls at electrodes 11 and 62 for both respond-to-target and suppress-to-target tasks. We conducted transformations to correct for possible violations of normality and homogeneity of variance. These analyses in parallel provided the same pattern of significance; therefore, only the analysis of raw data is presented here.

### *3.1. ERP analysis – Respond-to-target task*

We analyzed P3a amplitude in the respond-to-target task by using the peak ERP amplitude for each participant between 160–424 ms post stimulus onset at the frontal electrode. As expected, the planned comparison between the control group and ADHD-All indicated that the control group's peak amplitude was higher than the peak amplitude collapsed across ADHD subtypes, $F(1,60) = 10.05$, $p = 0.0024$, $\hat{\eta}^2 = 0.14$, power = 0.88. Also as predicted, a one-way ANOVA revealed a significant effect of levels of ADHD on peak ampli-

Table 2
Mean Amplitude and Latency for the P3a and the P3b on the Respond-To-Target Task

| Groups | P3a amplitude ($\mu$V) | P3a latency (ms) | P3b amplitude ($\mu$V) | P3b latency (ms) |
|---|---|---|---|---|
| Control | 3.29(2.59)$^{A}$ | 329(42.8) | 1.09(1.07) | 577(54.2) |
| ADHD-IA | 1.33(1.95)$^{B}$ | 307(58.9) | 0.77(0.90) | 538(63.2) |
| ADHD-HI | 1.31(1.16)$^{B}$ | 294(53.6) | 0.86(1.06) | 565(44.6) |
| ADHD-Combined | 2.00(1.66) | 290(57.9) | 0.59(1.23) | 542(65.8) |

*Note*$_1$. *SD* listed in parentheses.
*Note*$_2$. Significant differences between groups indicated with superscript upper case letters (A>B).

Table 3
Mean Amplitude and Latency for the P3a and the P3b on the Suppress-To-Target Task

| Groups | P3a amplitude ($\mu$V) | P3a latency (ms) | P3b amplitude ($\mu$V) | P3b latency (ms) |
|---|---|---|---|---|
| Control | 4.11(2.87) | 290(43.3) | 2.07(2.29) | 332(20.7) |
| ADHD-IA | 3.11(2.02) | 283(24.5) | 0.83(1.07) | 349(30.8) |
| ADHD-HI | 2.50(2.80) | 299(55.0) | 1.33(1.65) | 348(21.4) |
| ADHD-Combined | 3.04(1.96) | 267(42.4) | 1.26(0.73) | 354(14.1) |

*Note*$_1$. *SD* listed in parentheses.
*Note*$_2$. No significant differences found between groups.

tude, $F(3,60) = 3.79$, $p = 0.0147$, $\hat{\eta}^2 = 0.16$, power = 0.79. Post hoc pairwise comparisons using the Tukey HSD test ($CD = 1.78$, $\alpha = 0.05$) supported the prediction that the control group would have a significantly higher peak amplitude than the ADHD-IA (see Fig. 1). However, the comparison between the control group and ADHD-Combined did not reach significance, while the comparison between the control group and ADHD-HI was significant. We did not predict either of these two outcomes.

We analyzed P3a latency in the respond-to-target task using the peak ERP latency for each participant between 160–424 ms post stimulus onset at the frontal electrode. We did not make any predictions for this analysis. The planned comparison between the control group and ADHD-All indicated that the control group's latency was longer than the latency collapsed across ADHD subtypes, $F(1,60) = 4.42$, $p = 0.0398$, $\hat{\eta}^2 = 0.07$, power = 0.55. A one-way ANOVA, however, revealed no overall effect of levels of ADHD on peak latency, $F(3,60) = 1.78$, $p = 0.1605$, $\hat{\eta}^2 = 0.08$, power = 0.44.

For P3b amplitude analysis in the respond-to-target task, we used the peak ERP amplitude for each participant between 448–660 ms post stimulus onset at the posterior electrode. We did not succeed in replicating previous studies, for the planned comparison between the control group and ADHD-All was not significant, $F(1,60) = 1.27$, $p = 0.2647$, $\hat{\eta}^2 = 0.02$, power = 0.20. There were no effects of levels of ADHD on P3b peak amplitude (see Fig. 2), $F(3,60) = 0.60$, $p = 0.6191$, $\hat{\eta}^2 = 0.03$, power = 0.17, providing no support for the hypothesis that the control group would exhibit higher P3b peak amplitude than ADHD-IA and ADHD-Combined on the respond-to-target task.

Table 4
Mean Sensitivity (d') and Response Bias (log($\beta$)) on the Respond-To-Target Task

| Groups | $d'$ | log($\beta$) |
|---|---|---|
| Control | 4.52 (0.44)$^{A}$ | 1.94 (0.85) |
| ADHD-IA | 3.75 (0.69)$^{B}$ | 1.93 (0.63) |
| ADHD-HI | 3.85 (0.64)$^{B}$ | 1.34 (0.66) |
| ADHD-Combined | 4.04 (0.62) | 1.87 (1.02) |

*Note*$_1$. *SD* listed in parentheses.
*Note*$_2$. Significant differences between groups indicated with superscript upper case letters (A>B).

P3b latency in the respond-to-target task was analyzed using the peak ERP latency for each participant between 448–660 ms post stimulus onset at the posterior electrode. We did not formulate any predictions for this analysis, and the planned comparison between the control group and ADHD-All was not significant, $F(1,60) = 2.92$, $p = 0.0924$, $\hat{\eta}^2 = 0.05$, power = 0.40. There were no effects of levels of ADHD on peak latency at the posterior site for the respond-to-target task, $F(3,60) = 1.66$, $p = 0.1856$, $\hat{\eta}^2 = 0.08$, power = 0.41.

### 3.2. *Behavioral data analysis – Respond-to-target task*

We analyzed responses to targets and distracters in the respond-to-target task for each participant using

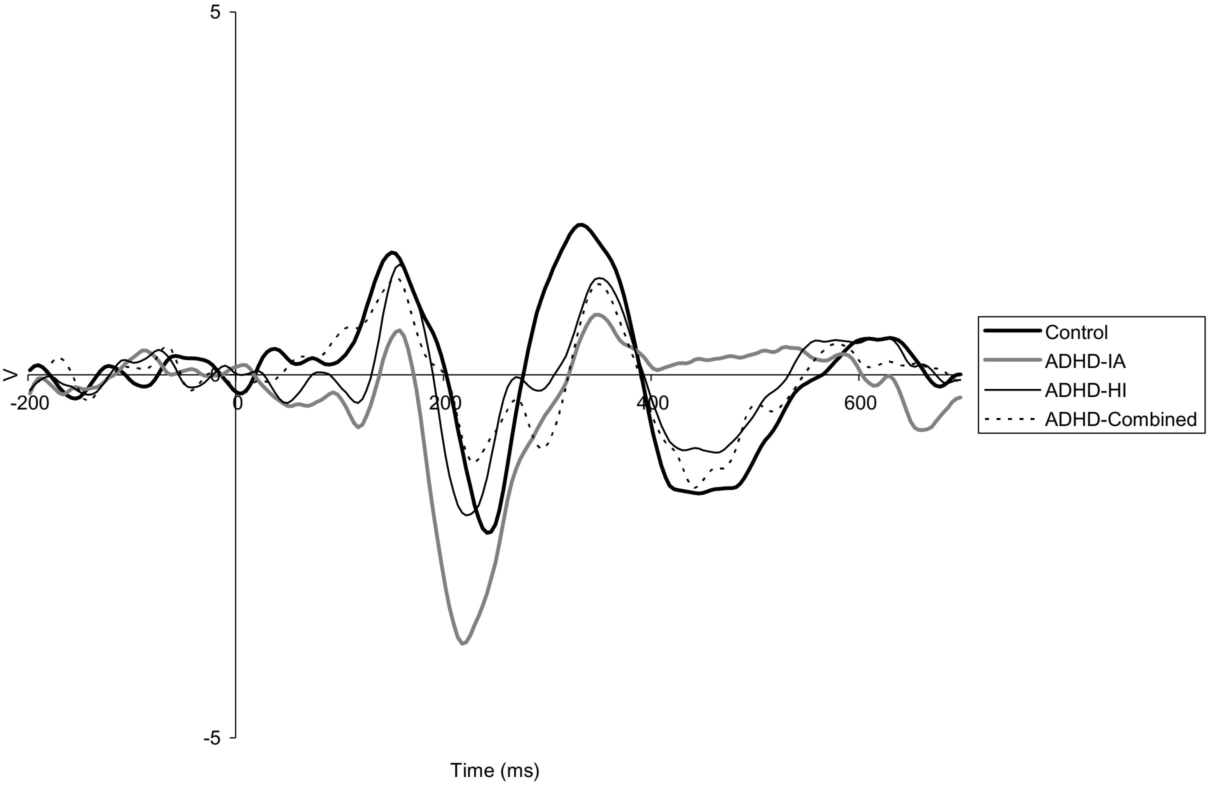

Fig. 4. Grand-average ERP waveforms for ADHD subgroups and controls at electrode 62 (comparable to Pz) on the suppress-to-target task.

Theory of Signal Detection. Table 4 shows the means and standard deviations of $d'$ and log($\beta$) for the ADHD subtypes and controls on the respond-to-target task. Results here coincide with prior studies, for the planned comparison between the control group and ADHD-All revealed that the control group had more signal sensitivity than the ADHD-All, $F(1,60) = 13.61$, $p = 0.0005$, $\hat{\eta}^2 = 0.18$, power = 0.95. A one-way ANOVA of the effect of ADHD on $d'$ was significant, $F(3,60) = 5.17$, $p = 0.0030$, $\hat{\eta}^2 = 0.21$, power = 0.91. Post hoc pairwise comparisons using the Tukey HSD test ($CD = 0.56$, $\alpha = 0.05$) partially supported the hypothesis. The control group had significantly more signal sensitivity than the ADHD-IA and ADHD-HI groups, but the hypothesis stated the control group would be more accurate than ADHD-IA and ADHD-Combined, not ADHD-HI. There were no log($\beta$) predictions formulated for this study, and the planned contrast between Control group and ADHD-All for the effect of ADHD on log($\beta$) (response bias) was not significant, $F(1,60) = 0.92$, $p = 0.3424$, $\hat{\eta}^2 = 0.01$, power = 0.15, as was the one-way ANOVA of the effect of ADHD on log($\beta$) among the four levels, $F(3,60) = 2.02$, $p = 0.1207$, $\hat{\eta}^2 = 0.09$, power = 0.50.

We used a one-way between-subjects ANOVA to analyze reaction times to targets in the respond-to-target task. Replication of studies finding controls to have faster RTs than individuals with ADHD was not achieved. The planned comparison between the control group and ADHD-All was not significant, $F(1,60) = 0.39$, $p = 0.5372$, $\hat{\eta}^2 = 0.01$, power = 0.09. A one-way ANOVA of the correct responses to the respond-to-target task also revealed no effect of ADHD on reaction time among the four levels, $F(3,60) = 0.53$, $p = 0.6656$, $\hat{\eta}^2 = 0.03$, power = 0.15.

### 3.3. *ERP analysis - Suppress-to-target task*

We analyzed P3a amplitude in the suppress-to-target task by using the peak ERP amplitude for each participant between 168–388 ms post stimulus onset at the frontal electrode. The planned comparison between the control group and ADHD-All showed the same pattern (see Fig. 3) as the respond-to-target task, where the control group's peak amplitude was higher than the

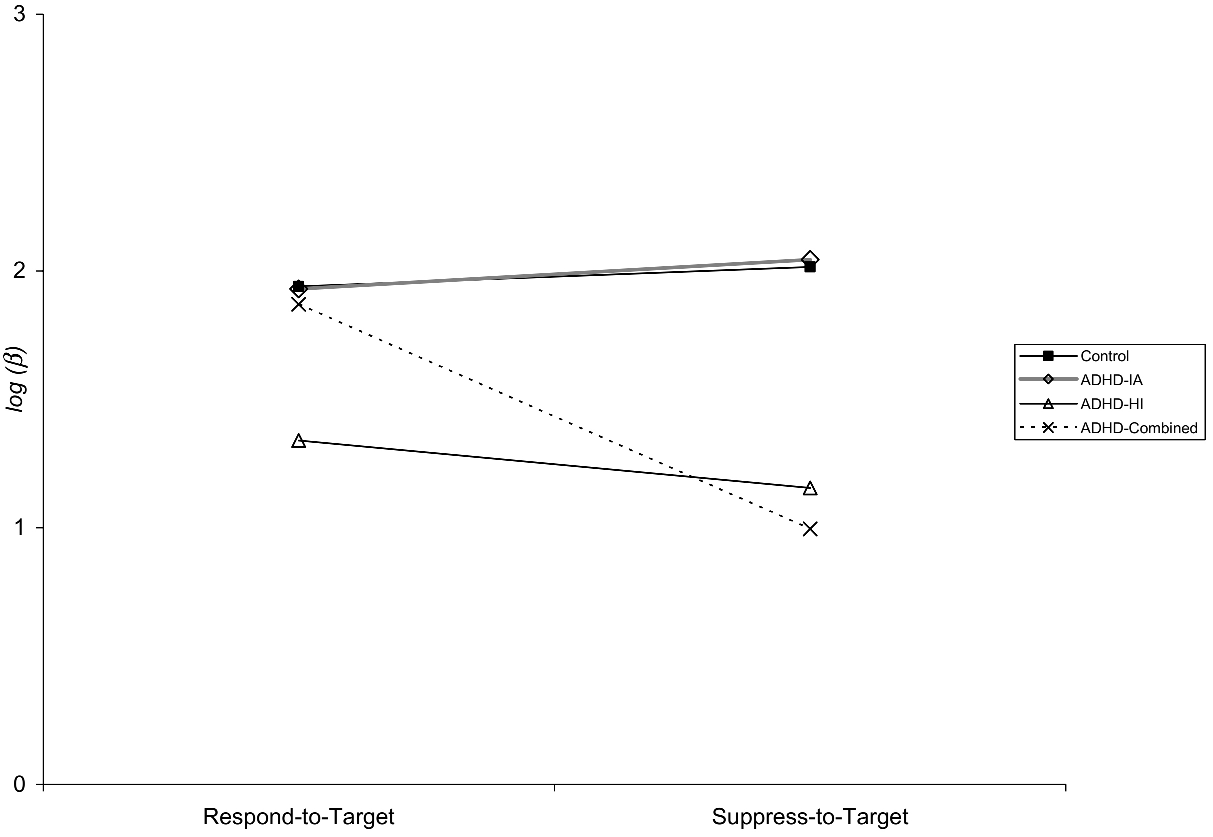

Fig. 5. Relation between ADHD and log($\beta$) (response bias) across respond-to-target and suppress-to-target tasks.

peak amplitude collapsed across ADHD subtypes, but this was only marginally significant, $F(1,60) = 3.02$, $p = 0.0876$, $\hat{\eta}^2 = 0.05$, power $= 0.41$, and did not replicate previous findings (although remains consistent with them). A one-way ANOVA did not support the hypothesis, revealing no effect of levels of ADHD on peak amplitude, $F(3,60) = 1.20$, $p = 0.3161$, $\hat{\eta}^2 = 0.06$, power $= 0.31$, even though the control group continued to display, as predicted, a higher peak amplitude than the ADHD subtypes as occurred in the respond-to-target task.

For P3a latency in the suppress-to-target task, we analyzed the peak ERP latency for each participant between 168–388 ms post stimulus onset at the frontal electrode. No predictions were made for P3a latency. The planned comparison between the control group and ADHD-All was not significant, $F(1,60) = 0.31$, $p = 0.5815$, $\hat{\eta}^2 = 0.01$, power $= 0.08$. There were also no effects of levels of ADHD on peak latency among the four groups, $F(3,60) = 1.67$, $p = 0.1831$, $\hat{\eta}^2 = 0.08$, power $= 0.42$.

P3b amplitude in the suppress-to-target task was analyzed using the peak ERP amplitude for each participant between 304–392 ms post stimulus onset at the posterior electrode. We did not find any effects of ADHD on peak amplitude at the posterior site for the suppress-to-target task (see Fig. 4), although the planned comparison between the control group and ADHD-All was marginally significant, $F(1,60) = 3.72$, $p = 0.0583$, $\hat{\eta}^2 = 0.06$, power $= 0.48$, which is consistent with prior studies. A one-way ANOVA revealed no significant differences between the four levels of ADHD, $F(3,60) = 1.53$, $p = 0.2168$, $\hat{\eta}^2 = 0.07$, power $=$ 0.38, and does not lend support to the hypothesis that the control group would exhibit a higher P3b amplitude than ADHD-HI and ADHD-Combined.

We analyzed P3b latency in the suppress-to-target task by using the peak ERP latency for each participant between 304–392 ms post stimulus onset at the posterior electrode. No P3b latency predictions were made for this study. The planned comparison between the control group and ADHD-All revealed that the ADHD-All group had a longer latency than the control group, $F(1,60) = 7.23$, $p = 0.0093$, $\hat{\eta}^2 = 0.11$, power $= 0.76$. The overall effect of ADHD on peak

Table 5
Mean Sensitivity (d') and Response Bias (log($\beta$)) on the Suppress-To-Target Task

| Groups | $d'$ | log($\beta$) |
|---|---|---|
| Control | 3.85 (0.59) | 2.02 (1.30)$^{A}$ |
| ADHD-IA | 3.32 (0.58) | 2.05 (0.83)$^{A}$ |
| ADHD-HI | 3.48 (0.76) | 1.15 (0.69)$^{B}$ |
| ADHD-Combined | 3.65 (0.49) | 1.00 (0.54)$^{B}$ |

*Note*$_1$. *SD* listed in parentheses.
*Note*$_2$. Significant differences between groups indicated with superscript upper case letters (A>B).

latency across the four levels approached significance, $F(3, 60) = 2.64$, $p = 0.0576$, $\hat{\eta}^2 = 0.18$, power = 0.62.

### 3.4. Behavioral data analysis – Suppress-to-target task

Responses to targets and distracters in the suppress-to-target task were analyzed for each participant using Theory of Signal Detection. Table 5 provides the means and standard deviations of $d'$ and log($\beta$) for the ADHD subtypes and controls on the suppress-to-target task. Findings here are consistent with previous research, for the planned comparison between the control group and ADHD-All revealed that the control group had more signal sensitivity than the ADHD-All group, $F(1, 60) = 4.34$, $p = 0.0414$, $\hat{\eta}^2 = 0.07$, power = 0.54. A one-way ANOVA of the effect of levels of ADHD on $d'$ (sensitivity), however, was not significant, $F(3, 60) = 2.21$, $p = 0.0965$, $\hat{\eta}^2 = 0.10$, power = 0.53, and did not support the hypothesis. The planned comparison between the control group and ADHD-All did not show any difference in log($\beta$) (response bias), $F(1, 60) = 0.20$, $p = 0.6546$, $\hat{\eta}^2 = 0.01$, power = 0.07. On the other hand, a one-way ANOVA of the effect of ADHD on log($\beta$) was significant, $F(3, 60) = 4.36$, $p = 0.0076$, $\hat{\eta}^2 = 0.18$, power = 0.85. Post hoc pairwise comparisons using the Tukey HSD test ($CD = 0.83$, $\alpha = 0.05$) indicated the ADHD-Combined and the ADHD-HI groups implemented a more liberal response strategy than the other two groups.

Reaction times to distracters (i.e., the errors of commission) in the suppress-to-target task were analyzed using a one-way between-subjects ANOVA. Results do not replicate previous studies where controls had faster RTs to distracters. The planned comparison between the control group and ADHD-All was not significant, $F(1, 60) = 3.65$, $p = 0.0587$, $\hat{\eta}^2 = 0.06$, power = 0.47. A one-way ANOVA of the errors of commission to the suppress-to-target task revealed no effect of ADHD on reaction time across the four levels, $F(3, 60) = 2.29$, $p = 0.0881$, $\hat{\eta}^2 = 0.11$, power = 0.55.

### 3.5. Interaction effects – Theory of signal detection

We noted during review of the analyses that an interaction exists between levels of ADHD and Go/NoGo tasks. We, therefore, conducted a post hoc comparison to further explore this interaction between behavioral performance across the levels of ADHD. A one-way repeated measures ANOVA of $d'$revealed that while all participants had more sensitivity to the respond-to-target task than the suppress-to-target task, $F(3, 60) = 18.97$, $p < 0.0001$, $\hat{\eta}^2 = 0.14$, power = 0.99, this difference did not interact with levels of ADHD, $F(3, 60) = 0.42$, $p = 0.7389$, $\hat{\eta}^2 = 0.01$, power = 0.13. However, a one-way repeated measures ANOVA of log($\beta$) indicated that the control group and ADHD-IA were equally conservative in both the respond-to-target and the suppress-to-target tasks, whereas ADHD-HI and ADHD-Combined exhibited a different pattern, responding more liberally in the suppress-to-target task than in the respond-to-target task (see Fig. 5), $F(3, 60) = 4.69$, $p = 0.0039$, $\hat{\eta}^2 = 0.11$, power = 0.89.

## 4. Discussion

The present study examined neurological and performance differences among the subtypes of ADHD and controls on a visual Go/NoGo task. The pattern was for the control group to elicit higher P3 amplitudes than the ADHD groups at both the frontal and the posterior electrode sites. Behaviorally, the ADHD-IA and ADHD-HI groups displayed less signal sensitivity on the attention-demanding respond-to-target task, while the ADHD-HI and ADHD-Combined groups were more liberal with their responses on the inhibition-demanding suppress-to-target task.

We partially achieved replication of previous studies. Results of the present study are consistent with those of Jonkman et al. [12], Kemner et al. [34], Kemner et al. [35], Miller et al. [3], Satterfield et al. [4], and Verbaten et al. [36] showing that the ADHD-All group exhibits a lower P3 amplitude than the control group in both respond-to-target and suppress-to-target tasks. These differences are significant in the respond-to-target task at the frontal site, not significant in the respond-to-target task at the posterior site, and

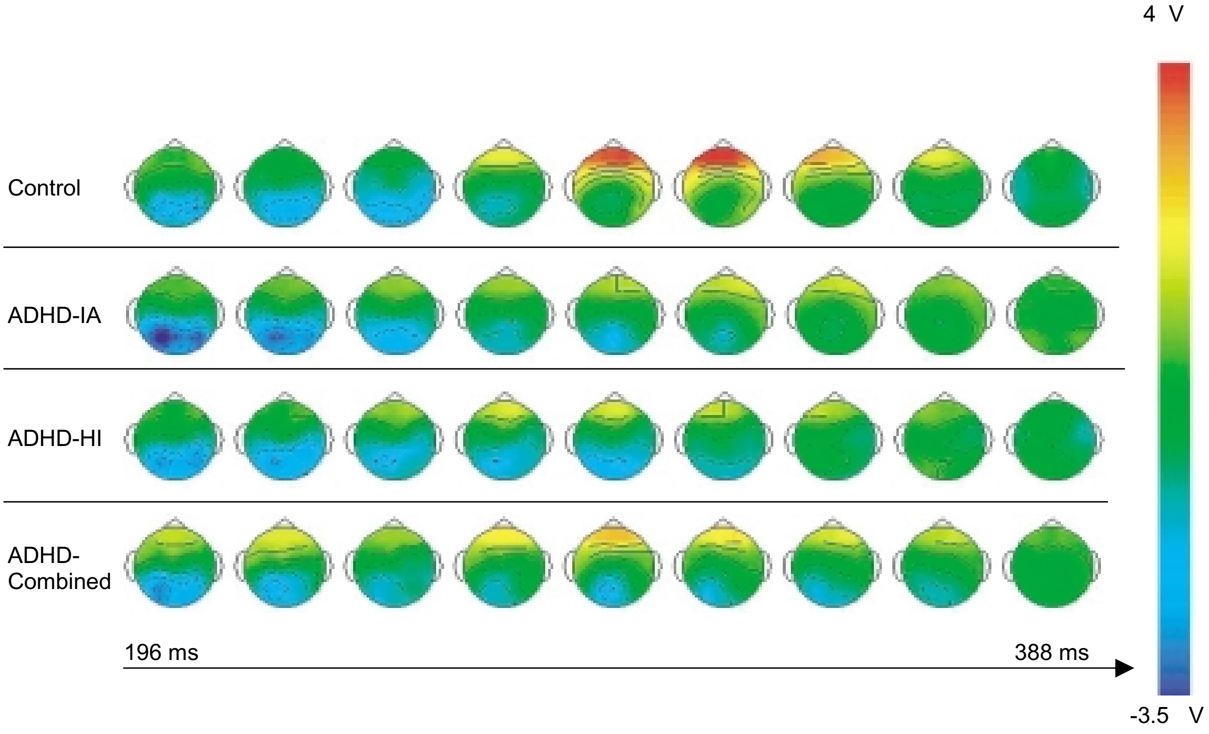

Fig. 6. Scalp topography associated with the P3a to the respond-to-target targets across ADHD subgroups and controls. There are 24 ms between frames.

marginally significant in the suppress-to-target task at both sites.

Results here do not replicate the findings by Jonkman et al. [12] where individuals with ADHD had longer reaction times than controls to targets in the respond-to-target task. Results are consistent with the works of Jonkman et al. [12] and Satterfield, Schell, and Nicholas [13] in that the ADHD-All individuals made more errors than controls in both respond-to-target and suppress-to-target tasks. While all the replications were not accomplished, the patterns found comparing the control group to the ADHD-All group in the present study, including the nonsignificant results, are consistent with these previous findings. Therefore, we consider results of the present study to reflect the population studied by other researchers. These partial replications are discussed where they relate to the hypotheses for the present study.

With respect to the subtypes, the first hypothesis for the present study stated that in the respond-to-target task, individuals in the ADHD-IA and ADHD-Combined groups would exhibit a smaller P3 peak amplitude than controls and that they would have less sensitivity to stimuli than controls. This hypothesis was partially supported, for the control group elicited a larger P3a peak amplitude and higher $d'$ than the ADHD-IA and ADHD-HI groups but not the ADHD-Combined group. This same pattern held true for the comparison between the control group and ADHD-All, which was expected from the studies by Jonkman et al. [12], Kemner et al. [35], and Satterfield et al. [4]. The suppressed P3a amplitude generated by the individuals with ADHD seen in Figs 1 and 2 indicates a dysfunction in initial attention allocation and in the attentional switch produced by the target as it differs from the passively neuronal trace of the distracters [30]. Controls were more effective at allocating their resources to the task, but the cognitively taxing task of identifying two dimensions of the stimuli (size and number) demanded more effortful attention than the individuals with ADHD could allocate [3]. The lower sensitivity to the targets in the respond-to-target task found in the ADHD-IA group, the ADHD-HI group, and the ADHD-All group indicates that individuals belonging to these groups had deficits in attention compared to controls, who are more successful at performing the task.

The second hypothesis stated that in the suppress-to-target task, individuals with symptoms of ADHD-HI

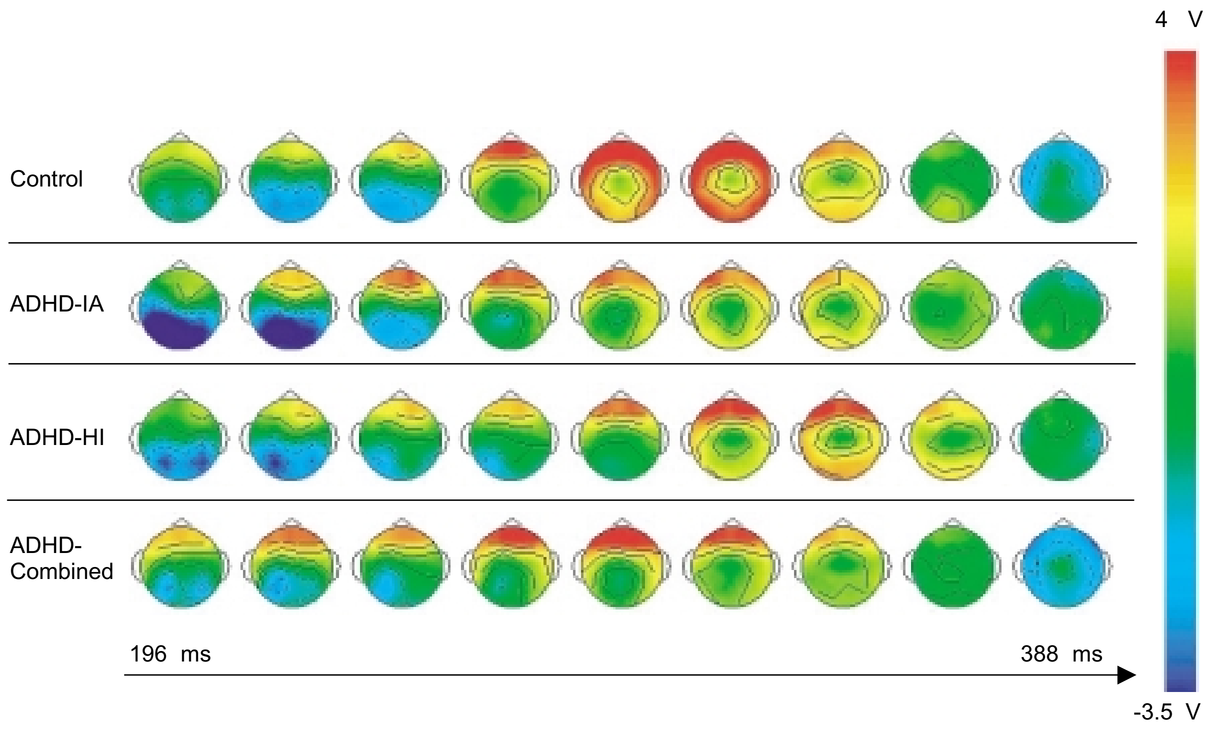

Fig. 7. Scalp topography associated with the P3a and P3b to the suppress-to-target targets across ADHD subgroups and controls. There are 24 ms between frames.

and those with symptoms of ADHD-Combined would exhibit a reduced P3 amplitude than controls and that they would also have less sensitivity to stimuli than controls. Results did not support the neurophysiological part of this hypothesis, for the four groups did not differ in their P3 amplitude, although the control group vs. ADHD-All comparison did approach significance. Though these findings were not significant, the trend remained consistent with studies by Jonkman et al. [12], Kemner et al. [35], and Satterfield et al. [4] who found controls to exhibit a higher P3 amplitude over the parietal electrode than individuals with ADHD. In addition, results show that controls were more sensitive to the suppress-to-target targets than the ADHD-All group, indicating a deficit in impulse control exhibited by the ADHD-All group.

The way we designed the current suppress-to-target task biased participants to press the key on the response box. Results indicate the individuals in the ADHD-HI and ADHD-Combined groups were pressing the key more so than accounted for by the demands of the task itself, further evidence of impulse-control deficits in these two groups, as predicted. The ADHD-IA group, on the other hand, due to the nature of their attentional deficit, they may have already been biased to not key press. Therefore, the demands of the suppress-to-target task did not bias them any more than the controls. These results provide support to Milich, Balentine, and Lynam's [5] conclusion that ADHD-IA and ADHD-Combined are best conceived as distinct and unrelated disorders, for here, the performance of the ADHD-Combined group resembled that of the ADHD-HI group and was not consistent with the performance of the ADHD-IA group.

We did not make any reaction time predictions for the levels of ADHD in this study, even though Jonkman et al. [12] stated that individuals with ADHD had slower reaction times than controls. However, prior to the present study, reasons did not exist to believe the subtypes would differ in their reaction times, and data analysis did not find any differences. This indicates that the errors of commission made by the individuals in the ADHD-HI and ADHD-Combined groups in the suppress-to-target task were not due to trying to be fast.

It is of interest to consider the relation between ADHD and the Go/NoGo tasks. ERP comparisons across tasks are not suitable here, for in this study, the respond-to-target task required more cognitive processing than the suppress-to-target task, which in turn lead to different P3 amplitudes. However, the reason for making the respond-to-target task harder was to lower the ceiling effect often found in similar tasks (e.g. [9]). Although participants analyzed two features of the respond-to-target targets (vs. one in the suppress-to-target task), the respond-to-target task was still a behaviorally easier task, for results show that all participants performed better on the respond-to-target task than on the suppress-to-target task. However, the types of errors made on the two tasks differ, for the interaction results indicate that individuals in the control, ADHD-IA, and ADHD-Combined groups made less key presses on the respond-to-target task than individuals in the ADHD-HI group, and in turn, individuals in the ADHD-HI group along with the those in the ADHD-Combined group made more key presses on the suppress-to-target task than the control and ADHD-IA groups (see Fig. 5).

It is also important to reiterate that the participants in this study were predominantly individuals with symptoms of ADHD, college students who in spite of possible attentional deficits were performing at a high enough level to pursue higher education. This could potentially explain the nonsignificant effects of ADHD on P3 amplitude found here. However, the ERP pattern displayed by the groups was consistent through both respond-to-target and suppress-to-target tasks at the frontal and posterior sites: controls elicited higher P3a and P3b peak amplitudes than the ADHD subgroups. Topographical maps in Figs 6 and 7 show the greater P3 amplitude in the frontal areas of the brain by the control group compared to the three ADHD-symptomatic subtypes in both respond-to-target and suppress-to-target tasks respectively. This is explained by the frontal lobes working harder in people whose frontal lobes work harder already. The individuals with symptoms of ADHD have a suppressed P3 during the respond-to-target task which is cognitively harder than the suppress-to-target task; therefore, their frontal cortex has to work harder than the controls' frontal cortex. The diminished P3 amplitude elicited by the ADHD-All in the suppress-to-target task could be due to a deficit in matching the current stimulus to the internal representation of the target. The extra effort the ADHD-All group made in this task suppressed the P3.

The nonsignificant ERP differences between controls and individuals in the ADHD-All group did approach significance. This encourages future studies to continue exploring the electrophysiological differences among ADHD subtypes using a medically diagnosed population with ADHD. The differences in behavioral responses in the present study were overt enough to be detected in this ADHD-symptomatic sample. The same should also hold true for individuals clinically diagnosed with ADHD.

## Acknowledgments

The authors express their gratitude to the following colleagues: John Richards, Stephanie Simon-Dack, Kristen Greene, Chris Rorden, Brad Smith, Jeff Shatz, & Jennifer Vendemia.
